# Cutting-edge communication and learning assistive technologies for disabled children: An artificial intelligence perspective

**DOI:** 10.3389/frai.2022.970430

**Published:** 2022-10-28

**Authors:** Katerina Zdravkova, Venera Krasniqi, Fisnik Dalipi, Mexhid Ferati

**Affiliations:** ^1^Faculty of Computer Science and Engineering, Ss. Cyril and Methodius University in Skopje, Skopje, North Macedonia; ^2^Department of Informatics, Faculty of Technology, Linnaeus University, Växjö, Sweden

**Keywords:** disabled children, assistive technologies, artificial intelligence, augmentative communication, alternative communication, voice recognition, speech recognition

## Abstract

In this study we provide an in-depth review and analysis of the impact of artificial intelligence (AI) components and solutions that support the development of cutting-edge assistive technologies for children with special needs. Various disabilities are addressed and the most recent assistive technologies that enhance communication and education of disabled children, as well as the AI technologies that have enabled their development, are presented. The paper summarizes with an AI perspective on future assistive technologies and ethical concerns arising from the use of such cutting-edge communication and learning technologies for children with disabilities.

## Introduction

According to the WHO (2021), more than a billion people worldwide experience some form of disability including almost 240 million children whose well-being is endangered. As also highlighted in a report of UNICEF (2021), the ageing of the world population, new illnesses and an escalating trend of chronic diseases further increase the amount of disabled people. They just need a little help to live and work independently and with dignity.

Aiming to assist loved ones, and often themselves, people started designing low-tech devices many centuries ago (Robitaille, 2010). Since the early sixteenth century, people with mobility difficulties have been using the walking cane as a tool that assists them to walk and stand stably (Amato, 2004). Although wheeled seats and furniture have been used for transporting disabled people since sixth century BCE, mass production of wheelchairs started in 1933, when the paraplegic Everest and his friend Jennings designed a metal foldable wheelchair that used the X-brace design they patented as a “construction for collapsible invalids' wheelchairs” (Woods and Watson, 2004).

Glasses for partially sighted people were invented in the seventeenth century (Rosenthal, 1996; Lee, 2013). At the same time, ear trumpets, passive amplifiers that collect sound waves, and direct them into the ear channel, replaced the cupped hand, the popular method for hard of hearing used since Roman Emperor Hadrian's era (Valentinuzzi, 2020). These two sensory tools are the origins of modern assistive devices and technologies that support a more independent life for people with disabilities (Robitaille, 2010).

Blind people were unable to independently learn and study until the beginning of the nineteenth century. At that time, Louis Braille, who was blind since his early childhood, modified the military purpose language created by Charles Barbier de la Serre and invented the Braille alphabet, which is still among the most widely used reading media for the blind (Jiménez et al., 2009). In 1935, Agatha Christie's novel The Murder of Roger Ackroyd was recorded, initiating the transition of the printed toward the so called talking books (Philips, 2007). Electronic text-to-speech synthesizers were unveiled just a few years later, enabling blind people to consume written content by leveraging their enhanced hearing ability (Ohna, 2010).

The first electronic assistive technology ever developed was the Akouphone, a portable hearing device with carbon microphone and earphones (Kenefick, 2009). Although communication based on hand movements is mentioned in Plato's Cratylus, a Socratic' dialogue from his middle period as early as the fifth century BCE (Cratylus, 2022), the first sign language in wide use was the Old French Sign language, which originated in the eighteenth century and became the basis of American Sign language (Reagan, 2021).

All these developments and tools significantly improved the condition of people with disabilities in their daily communications, although their comprehensive education was still challenging. To ensure the fundamental human right to education and avoid the discrimination due to disability, in 2018 WHO launched the global cooperation on assistive technology (GATE) initiative (Boot et al., 2017). The only goal of GATE is “to improve access to high-quality affordable assistive products globally” (WHO, 2018).

Artificial Intelligence (AI) is the driving force of most assistive products, supporting people with different disabilities to keep and improve their education and everyday activities (Zdravkova, 2022). It enabled the creation of simulated environments that also include augmented and virtual reality. AI-supported tools improve visual tracking skills, help students with social disabilities and improve time-management skills.

The advantage of AI technologies over non-AI technologies used to date is the speed and precision they provide in analyzing and deciphering complex communication, expression, and visual behaviors. For example, applications that incorporate gesture-based text prediction in conjunction with AI are very useful for categorizing the most likely words from gestures and transforming them into meaningful sentences that support people with hearing impairments (Cheng and Lai, 2020). Other examples include machine and deep learning, which can improve substantially EEG-based brain-computer interfaces that help people who are unable to move gain independence (Sakti et al., 2021).

In this study, we present the findings of a narrative literature review that collected relevant literature published during the last ten years (Baumeister, 1997). The first step in this method pertains to conducting a preliminary search of the literature. For this reason, we constructed a search string to query the Scopus research database. In this endeavor, we have included papers only written in English that highlight evidence related to the impact of AI on modern assistive technologies for children with disabilities. As a next step, we analyzed and refined the explored topics from the relevant papers by subsequently identifying the employed AI technique to support and enhance the functionality of the assistive technologies. After a careful review, we classified the AI techniques into four different clusters, namely, augmentative and alternative communication (AAC), machine and deep learning (ML and DL), natural language processing (NLP), and Conversational AI.

The remainder of the paper is organized as follows: in the next section, a variety of disabilities will be explored and the cutting-edge assistive technologies that support communication and education of young children will be announced in line with AI technologies that enabled their creation. This section will be followed with a brief explanation of AI algorithms and techniques behind the announced assistive technologies including the futuristic ones. The paper will conclude with an AI perspective of future assistive technologies and ethical concerns that arise from the use of cutting-edge communication and learning assistive technologies intended for disabled children.

## Cutting edge assistive technologies

Unhindered communication is the key prerequisite of quality education (Dhawan, 2020). If a student cannot listen to what a teacher presents and school mates talk about, or cannot see the visual content that supports the lectures and the assignments, then the effect of instructional behavior exhibited even by the most skilled teachers is reduced. Lack of attention, cognitive and intellectual preparedness to comprehend the school curriculum is an additional problem. Sometimes, perfect sight, hearing and intellectual abilities are obstructed by motor disabilities, which slow down or disable students' ability to write. Cutting-edge assistive technologies can mitigate many of the above-mentioned problems (Shinohara and Wobbrock, 2011).

According to American Speech-Language-Hearing Association (ASHA), communication disorders cover the impairments “in the ability to receive, send, process, and comprehend concepts or verbal, non-verbal and graphic symbol systems” (American Speech-Language-Hearing Association, 1993). They are classified into speech disorders, language disorders, hearing disorders, and central auditory processing disorders (American Speech-Language-Hearing Association, 1993).

**Figure 1:**
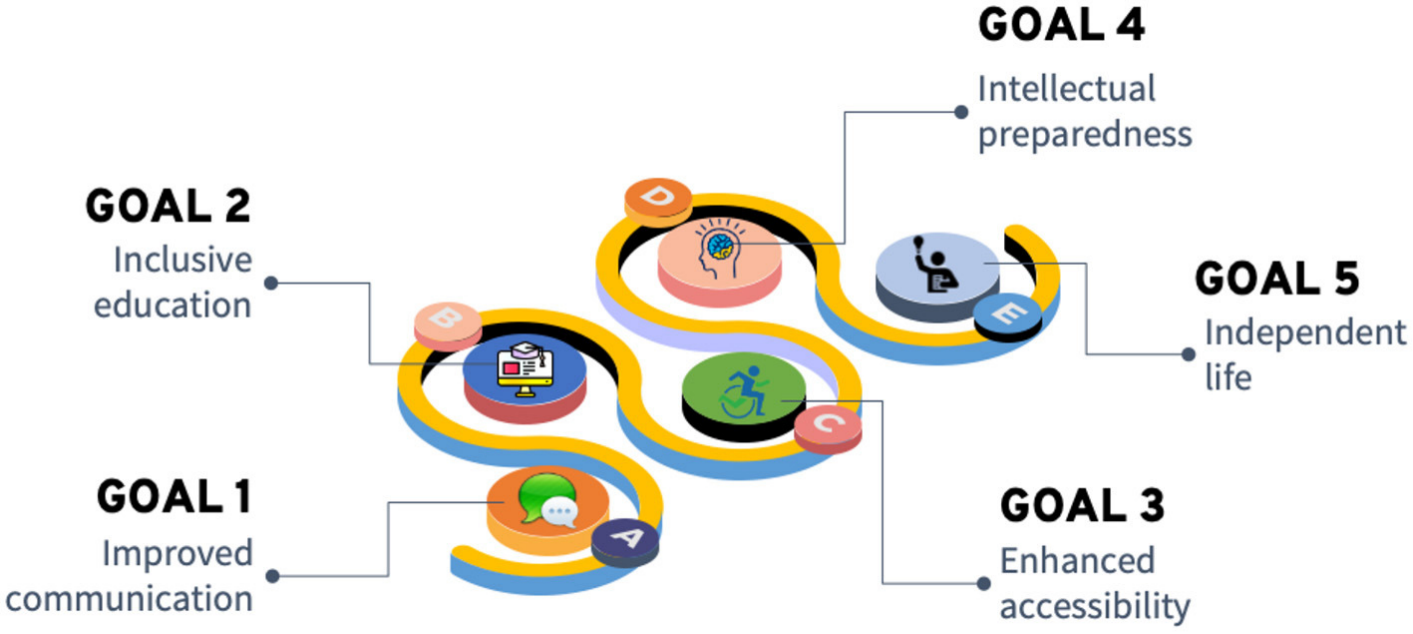
AI enabled goals to support children with disabilities.

The modern assistive technologies empowered by AI can significantly contribute to achieving important goals to support and provide new possibilities for children with disabilities. In Figure 1 we illustrate such goals, which include *improved communication, inclusive education, enhanced accessibility, intellectual preparedness*, and culminating with *independent life*.

As mentioned earlier, the AI techniques were classified into four broad clusters: AI methods and algorithms that support AAC; ML and DL; NLP; and Conversational AI – speech and voice recognition, which are further divided into silent speech interface (SSI), speech recognition (SR), visual speech recognition, and voice recognition. These four clusters should not be mixed with the AI goals that according to Russel and Norvig (http://aima.cs.berkeley.edu/) embrace: problem solving; reasoning; knowledge representation; planning; machine learning; communication, perceiving and acting. Deep learning and natural language processing are important AI subsections and tools, while conversational AI is an umbrella term of so called affective computing, which is an interdisciplinary field that relies on AI. AAC is not AI per se. Similarly to affective computing, it heavily relies on sophisticated AI. Although AI heterogeneous, the division was made according to the needs of assistive technologies, thus they are a symbiosis of AI methods and major trends in assistive technologies.

Table 1 lists on the first column the senses or the human ability being affected. For each sense then a disorder is listed (column two) followed by the appropriate assistive technologies (column three) designed to easier overcome the challenges associated with the disorder. In the table's last two columns, we list AI techniques and AI algorithms and methods respective of the listed assistive technologies. The main criteria when selecting the listed disorders was their impact on impaired communication and the availability of Scopus studies describing the appropriate assistive technologies. If multiple studies introduced the same assistive application, priority was given to the one that in more detail represents an implementation of the AI technologies, algorithms, and methods.

**Table 1:** Assistive technologies aligned to impaired communication disorders.

**Affected ability**	**Disorder**	**Assistive technology**	**AI techniques**	**AI algorithms/methods**
Speech	Apraxia of speech	Tabby Talks for administration of speech therapy (Shahin et al., 2015)	AI based AAC NLP Conversational AI (SRVR)	MLP classifier PVer VAD
	Dysarthria	Italian project (under construction), Vivoca (Mulfari et al., 2021; Lokitha et al., 2022)	AI based AAC ML Conversational AI (SR)	CNN SVM TensorFlow
	Mutism	BridgeApp (Samonte et al., 2019)	AI based AAC	Bayesian inference
	Stuttering (stammering)	Fluent (Manjula et al., 2019)	AI based AAC ML and DL	AANAO
	Articulation disorder	LipNet, MIT Media Lab and SottoVoce (Li et al., 2022)	ML and DL Conversational AI (SSI, SR, VR, VSR)	CNN DNN LSTM NN RNN SCNN
Sight	Blindness	JAWS, Electronic Braille, Emacspeak, ORCA, VoiceOver, Windows-Eyes (Wahidin et al., 2018)	NLP ML and DL	BLSTM HMM Hybrid CNN and RNN RNN
Hearing	Deafness	AVA, Live Transcribe, Rogervoice, Signly (Ridha and Shehieb, 2021; Kumar et al., 2022)	AI based AAC ML and DL NLP	AR BERT CNN HMM GMM RNN
Hearing and Sight	Dual sensory loss (Deafblindness)	Communication gloves, Tactile boards (Ozioko et al., 2018; Theil et al., 2020)	AI based AAC ML and DL NLP Conversational AI (SpR, SR, VoR)	HC STB
Speech and Writing Understanding spoken and written language	Aphasia	Voice recognition system (Ali et al., 2021)	AI based AAC ML and DL NLP Conversational AI (VR)	k-Nearest Neighbors Naïve Bayes
Navigation	Spinal cord injury	ColorCode (Daly, 2022)	AI based AAC	Bayesian learning

The following AI algorithms and methods were used to develop the assistive technologies shown in the table: adaptive optimization based on artificial neural networks (AOANN), augmented reality (AR), Bayesian learning, Bidirectional Encoder Representations from Transformers (BERT), Bidirectional long short-term memory (BLSTM), Convolutional neural networks (CNN), Deep neural networks (DNN), Gausian Markov model, Haptic communication (HC), Hidden Markov model (HMM), Hybrid CNN and RNN, k-Nearest Neighbors, Multi-layer perceptron (MLP) classifier, Naïve Bayes, Pronunciation verification, Recurrent neural network (RNN), Spatiotemporal convolutional neural network (SCNN), Speaker recognition (SpR), Speech-to-Braille, Speech-to-text (STT), Support vector machines (SVM), TensorFlow, Text-to-speech (TTS) and Voice activity detection (VAD).

AI algorithms presented in the table were selected in three stages:

a) Determination of the most frequent communication disorders covered by American Speech-Language-Hearing Association (ASHA, https://www.asha.org), mental disorders from World Health Organization (https://www.who.int), as well as learning difficulties listed in the UNESCO learning portal (https://learningportal.iiep.unesco.org/);
b) Selection of the relevant scholar articles that present AI-based assistive technologies created to support this communication and learning disorders;
c) Thorough examination and extraction of AI methods, algorithms and techniques and their clustering.

Next subsection introduces them briefly by presenting a short explanation of each of the implemented AI algorithms and the assistive technologies that were developed by implementing those algorithms. Then, AI-based assistive technologies from all four clusters are also introduced and illustrated.

### AI algorithms and assistive technologies for communication and learning impaired students

The list of the algorithms in the previous section was alphabetically ordered. The introduction of AI algorithms within the subsection will start with artificial neural networks, which are the most widely used computational model in machine learning and the heart of deep learning. The list will continue with the learning algorithms, then it will introduce speech and text conversions and end with haptic technology.

Since the mid 1980s, artificial neural networks have become a very powerful model that enables excellent prediction and learning of many patterns that cannot be explicitly presented. According to Pai (2020), three types of neural networks contribute to deep learning: artificial neural networks (ANN), CNN, and RNN. ANNs are feedforward networks capable of learning nonlinear functions. Their limitations can be overcome by creating building blocks (CNN) or by adding a recurrent connection on the hidden state (RNN). CNNs are particularly valuable for image processing, while RNNs solve problems with time series, text and audio data (Pai, 2020). Combination of CNNs and RNNs, sometimes called hybrid CNN and RNN proved its efficiency for word prediction (Goulart et al., 2018). Spatiotemporal convolutional neural networks are a special type of CNNs capable of extracting spatial-temporal features. They are very efficient for sign language recognition (Li et al., 2022). More complex neural networks with many hidden layers that employ more sophisticated mathematical models belong to deep neural networks.

Bidirectional Encoder Representations from Transformers (BERT) is a language representation model that is trained on unlabeled data over different pre-training tasks using multi-layer bidirectional transformer encoders (Devlin et al., 2018). BERT is embedded into Google Search for over 70 languages. It has been successfully used in several assistive technologies, particularly for speech recognition (Brunner et al., 2020) and speech completion (Tsunematsu et al., 2020). Model performance of classification problems of sequential data can be improved by BLSTM. It is particularly useful for developing assistive technologies for people with visual impairment (Wahidin et al., 2018).

Artificial neural networks adaptive optimization is an effective classifier that was used to predict the disfluencies in speech signals of people with stuttered speech (Manjula et al., 2019). It adaptively optimizes network architecture using the artificial fish swarm optimization method, which implements stochastic search (Manjula et al., 2019).

k-Nearest Neighbors is non-parametric, supervised learning classifier, which is widely used for computing the distances from the test example to all stored examples. It was abundantly used for classification and regression in the voice recognition systems (Ali et al., 2021).

Support vector machines are supervised learning models used for pattern classification (Cortes and Vapnik, 1995). They were used for developing the smart voice conversational assistant (Lokitha et al., 2022).

The term Bayesian learning has been interchangeably used with Bayesian inference. It produces a probability distribution using the Bayes' theorem to predict the value of an unknown quantity (Neal, 2012). Naïve Bayes classifiers are a simple class of Bayesian networks capable of efficient classification and prediction. Bayesian learning and naïve Bayes have been successfully used to develop BridgeApp, an assistive mobile application that assists communication between people that are deaf and mute (Samonte et al., 2019).

Hidden Markov model is a statistical Markov model that supports modeling of an observable process using unobservable states. Gausian hidden Markov models expect that the observation probability distribution is Gausian (or normal). These two finite-state models establish correlations between adjacent symbols, domains, or events, which is crucial for speech recognition.

Speaker, speech and text recognition are crucial to enable smooth communication with and among people with more severe hearing and visual impairment. Speaker and speech recognition identify words spoken aloud and convert them into readable text presented with written or Braille alphabet (Benzeghiba et al., 2007). They implement almost all AI algorithms to develop various assistive technologies, such as AVA, Jaws or RogerVoice (Zdravkova and Krasniqi, 2021). Assessment of speech in TabbyTalks (Shahin et al., 2015) implements (VAD, Sohn et al., 1999).

Augmented reality (AR) is an umbrella term that embraces interactive experience by integrating 3D virtual objects into a 3D environment in real time (Azuma, 1997). AR has been widely used in various assistive technologies, including the popular live captioning system AVA (https://www.ava.me/), which enables deaf and hard-of-hearing to read the spoken lectures.

People with dual sensory loss can rely on haptic communication (HC), that enables tactile communication and interaction via the sense of touch (Ozioko et al., 2018). Many assistive technologies use the open source software library TensorFlow library (www.tensorflow.org), which was developed by Google.

### AI-based assistive technologies

In all the presented assistive technologies, AI has been abundantly used. Augmentative and alternative communication methods proved their significant role in at least half of them. Assistive technologies related to hearing or visual deficiency are developed using neural natural language processing algorithms, which are a symbiosis between natural language processing and deep learning. The predominance of neural and deep networks is also obvious, proving that new assistive technologies are machine learning powered.

AAC is the best assistive remedy for intelligibility, which is mildly or more severely disrupted by speech disorders, including: aphasia, apraxia of speech, articulation disorder, cluttering, dysarthria and stuttering (Kent, 2000). It can also be affected by cognitive problems, such as autism spectrum disorder (ASD), dyslexia and Down syndrome (Deb et al., 2022; Krasniqi et al., 2022), and by motor disabilities, for example cerebral palsy, multiple sclerosis, and Parkinson's disease (Stipancic and Tjaden, 2022). Assistive devices and technologies that are used to improve intelligibility are among the most prominent AAC devices and technologies (Norrie et al., 2021). Although the success of high-tech AAC is still limited, mainly due to “infrastructure, policy, and recruitment deficits”, their advancement is inevitable and they will soon “serve as mediator between teacher, aided communicator, and their assistive technology” (Norrie et al., 2021). It is expected that in the near future, AAC devices will be combined with non-invasive methods of access to the brain-computer interface, which will revolutionize communication (Luo et al., 2022). However, without powerful machine learning and neural imaging technology, this transformation will never become true.

Within most of the reviewed devices and technologies, machine learning is developed alongside deep learning. It is particularly powerful in the technologies related to speech and sound production, as well as with spoken and written language understanding (Jobanputra et al., 2019). Neural network brain-computer interfaces (NNBCI) have a potential to reduce disability by translating neural activity into control of assistive devices (Schwemmer et al., 2018).

Speech and voice recognition will never be possible without artificial intelligence (Singh et al., 2018). All modern deep learning techniques, including BERT (Brunner et al., 2020) contribute to better speech, voice and speech recognition (Amberkar et al., 2018). Non-invasive brain-computer interfaces emerge in this area, leading to much better performance compared to traditional systems that process auditory and visual information (Brumberg et al., 2018). United with deep learning methods and architectures, they “boost classification performance” of algorithms for computer vision and natural language processing (Singh et al., 2018).

Most of the tools introduced in this section have been developed to accommodate the needs of students starting from elementary education to the college level. On some occasions, they can be applicable even for elderly people. While the concept of AI technologies presented so far was mainly related to applications in the education sector, AI has also the potential to improve health and well-being of elderly people. Some forms of AI assistive technologies such as autonomous robots, AI-enabled health applications, voice-activated devices and intelligent homes could tackle the key aging related challenges.

Cutting-edge technologies should be carefully designed and should consider privacy and content. While children with disabilities are more familiar with the use of phones, which facilitate the design and implementation of AI technologies, more mature people need assistance for an independent life. To provide AI technologies to elderly people, designers and programmers of these technologies should implement some considerations tailored to their needs: ensure the participation of elderly people for development of AI technologies, cross-age data collection, investments in digital infrastructure, increased research to understand new uses of AI and how to avoid bias (WHO, 2022).

To conclude, cutting-edge technologies significantly improve communication and learning of people with disabilities, and particularly of young children who were born with technology and do not hesitate to use them without any effort and resistance. Next section will try to prove this claim by researching more thoroughly the four clusters announced in the introduction of this section.

## AI technologies that support communication and learning assistive technologies

### Augmentative and alternative communication

Certain people with disabilities cannot use speech as their primary means of communication and need therefore to find an alternative way or specific techniques to express themselves. The idea of Augmented and Alternative Communication (AAC) is to use all the abilities that a person has, in order to compensate for the impairment of the verbal communication capacity (Chirvasiu and Simion-Blândă, 2018). In other words, the AAC system provides effective communication to maximize quality of life. There exist various types of AAC that can be chosen depending on the individual's skill and communication needs (Beukelman et al., 2013). The ACC systems are classified as unaided and aided. Unaided systems do not require a physical aid or tool (e.g., facial expressions, sign language). Aided systems, on the other hand use materials or tech devices which are categorized as:

– Low -tech devices (symbol boards, choice cards, communication books)
– High-tech devices (AAC apps on mobile devices, speech-generating devices or communication devices)

The rapid development of AI has recently opened up new ways to address more and more complex challenges, such as for instance, helping people with complex communication needs to overcome barriers (Delipetrev et al., 2022).

AI powerful tools have the capability of transforming AAC systems such as low-tech with words and symbols and high-tech with computers that employ a human voice for output (Beukelman et al., 2013). Below, AI technologies embedded in the AAC are presented with an analysis of how these tools are being developed and deployed to meet the diverse needs of users.

As part of the UNICEF's Innovation Fund Investments in Skills and Connectivity (OTTAA Project: AI Algorithms for Assistive Communications, 2022) a platform (OTTAA project) is developed, which is considered to be the first augmentative and alternative communication (AAC) platform that uses a combination of powerful AI algorithms (NLP and ML) and pictogram-based communication code to create sentences and communicate effectively. OTTAA platform allows speech-impaired people to communicate and express themselves using a simple three-tap interaction. Using appropriate pictogram-based communication and AI algorithms children with disabilities will have the opportunity to communicate better and faster (OTTAA Project: Accessible Communication for Children with Disabilities, 2022). In order to encourage more diversity and options for users, the algorithm is trained constantly by analyzing more than 1.8 million sentences previously created by other users. OTTAA is an open source platform that encourages people to participate in improving the source code, in this way it creates an environment where everyone feels that they are contributing.

Image recognition technologies are considered pivotal in inclusive education to make learning accessible and effective. GoVisual app is a program converting photos and videos into literacy and communication opportunities on an iPhone or iPad (GoVisual™, 2022). This innovative approach combines computer vision using the image recognition technology (collecting photographs and videos), the NLP tools to help in story creation, and finally the ML to help identify objects and shapes for ease of programming (Tintarev et al., 2016). Combination of these three techniques creates a potential of independence and self-determination for children with disabilities in their school environment.

HearMeOut app is an application which incorporates both gesture-based text prediction and pictogram-based augmentative and alternative communication using AI. The application uses Natural Language Conversation (Srivastava, 2021) enabling the disabled users to engage in conversation using Speech Recognition. It also uses a word level sign language dataset (Li et al., 2022) to categorize the most probable words from the gestures of the impaired person captured using a camera and then transforms them into the most meaningful and possible sentence using state-of-the-art algorithms. Another positive aspect to this approach is the security concerns; the application does not store the user data because of the continuous process of input and output without storage, which minimizes the chances of any data leakage.

“Fluent” is an AI Augmented Writing Tool which assists persons who stutter to identify words that they might struggle pronouncing and presents a set of alternative words which have similar meaning but are easier to pronounce. The overall landscape of AI-based writing tools is typically comprised of NLP based software systems (Ghai and Mueller, 2021) as in this app but in addition it uses AL (active learning, that is the subset of machine learning; Settles, 2011) to identify whether it can help learn the unique phonetic patterns that an individual might struggle pronouncing. The app does not intend to improve the stutter condition but helps camouflage it.

### Machine and deep learning

The recent increase in computing capabilities has enabled ML algorithms to further enhance the functionalities of assistive technologies. The incorporation of ML into eye tracking technology can contribute to the development of smarter assistive systems for people with disabilities (Koester and Arthanat, 2018; Yaneva et al., 2020).

In a study conducted by Valliappan et al. (2020), ML is leveraged to demonstrate accurate smartphone-based eye tracking without any additional hardware. The study results highlight the utility of smartphone-based gaze for detecting reading comprehension difficulty and confirms findings from previous studies on oculomotor tasks. Another work was conducted by Deepika and Murugesan (2015) to facilitate the interactions between computers and people with special needs using eye tracking technology. The performance accuracy of the proposed system under good lightning conditions was 97%.

Additional research efforts are concentrated on people with motor disabilities for a hands-free computer interaction (Šumak et al., 2019), to measure the variation between fixations and saccades using K-means analysis (König and Buffalo, 2014), and for training purposes to control eye gaze *via* VR (Zhang and Hansen, 2019).

Research advances in machine and deep learning have also contributed to improved electroencephalogram (EEG) decoding and target identification accuracy. In this perspective, visual evoked potential (VEP) based brain-computer interface (BCI) systems are widely explored, mainly due to low user training rate (Waytowich et al., 2016). One research involving people with motor and speech disabilities to evaluate a new monitor for generating VEP for daily BCI applications is conducted by Maymandi et al. (2021). The target identification in this study was performed using DNN. Moreover, DNN have become a useful approach to improve classification performance of BCI systems using EEG signals (Kwak et al., 2017; Craik et al., 2019).

A framework for brain electrical activity-based VEP biometrics is proposed by Palaniappan and Mandic (2007). In this work, in order to improve the classification accuracy, authors utilized k-Nearest Neighbors (kNN), Elma Neural Network (ENN) classifiers and 10-fold cross validation classification.

Video accessibility is of paramount importance for blind and visually impaired individuals for education and other purposes. Computer vision applications show promising results on removing the accessibility barriers, especially toward helping blind people to better sense the visual world. The most recent applications of ML in computer vision are object detection and classification and extraction of relevant information from images or videos.

A machine learning based approach to video description by automating video text generation and scene segmentation is proposed by Yuksel et al. (2020). The quality of the video descriptions generated through this system compared to the human-only condition resulted in being rated higher by blind and visually impaired users. A multimodal comprehensive accessibility framework to generate accessible text and tactile graphics for visually impaired people is suggested by Cavazos Quero et al. (2021). The framework uses machine learning, i.e., image classification technique to classify various kinds of graphics and applies simplification methods to the graphics category. Recently, interactive machine learning (IML) is utilized to support interface design through workshops with disabled users (Katan et al., 2015). This work demonstrates IML's potential significance as a design tool, expediting the design process by allowing the swift mapping of participant observations into prototypes.

### Natural language processing techniques

Screen and text magnifiers are very useful solutions for low vision people, enabling them to read and adjust text. They zoom in the whole screen or a selected section of the screen without any AI techniques utilized. On the other hand, screen readers, Braille displays, and speech recognition software assistive technologies for blind people are completely AI based (Choi et al., 2019).

Screen readers are a compulsory part of all the popular learning management systems: Blackboard Learn, Brightspace D2L, Canvas LMS and Moodle (Zdravkova and Krasniqi, 2021). JAWS is embedded in all of them with Chrome, NVDA, TalkBack, ORCA and VoiceOver as alternative text-to-speech plugins (Oh et al., 2021). As a standalone application or part of Web applications, screen readers support image and touchscreen accessibility (Oh et al., 2021). Apart from speech, they enable non-speech audio, vibration, tactile and force feedback (Oh et al., 2021).

Screen readers consist of two components: optical character recognition (OCR) that recognizes text, images and mathematical expressions; and text-to-speech (TTS), which delivers that content in the form of speech (Suzuki et al., 2004). They can be additionally enhanced by a machine translator enabling language localization (Suzuki et al., 2004).

OCR passes through several phases, three of which are AI-powered: image pre-processing that removes the potential distortions and transforms the image into light and dark areas; intelligent character recognition that compares scanned characters with the learned ones; and post-processing that corrects the errors (Chaudhuri et al., 2017). Past OCR algorithms for text recognition were realized with pattern recognition and ML techniques (Rao et al., 2016). Recent algorithms unite the following soft computing constituents: fuzzy sets, artificial neural networks, genetic algorithms, and rough sets (Rao et al., 2016).

Image recognition is enhanced by image captioning tools, which generate textual description of visually presented objects using template-based, retrieval-based and neural networks-based methods (Wang et al., 2020). Template-based method is a statistical modeling method that uses HMM, Maximum Entropy Markov Models, and Conditional Random Fields to recognize and machine learn the patterns (Wang et al., 2020). The retrieval-based method measures the visual similarity between a new image and an already interpreted image and generates a human-level sentence (Wang et al., 2020).

Recognition of mathematical expressions and formulas, including the handwritten ones consists of character recognition and structure recognition (Veres et al., 2019). Both recognition tasks depend on various ML methods, such as Bayesian inference, fuzzy logic, and neural networks (Veres et al., 2019).

At the end of the OCR part, information is stored as data or text waiting to be converted into voice. The conversion is done by TTS systems, which is a pure natural language processing task (Mache et al., 2015). It consists of text analysis, phonetic analysis, prosodic analysis, followed by speech synthesis (Adam, 2020). Deep neural networks are the most frequently used methods of modern TTS systems that successfully predict the acoustic feature parameters for speech synthesis (Adam, 2020).

Screen readers, which deliver content into speech or auditory signals, are not suitable for deafblind people. The best alternative are text to Braille translators, which “interpret letters and figures through a tactile display” (Gote et al., 2020). Refreshable Braille displays are fully supported by Blackboard Learn and partially supported by Brightspace D2L (Hsu, 2020). They use AI techniques and methods only during OCR phase (Gote et al., 2020). In contrast, AI is the key factor in the opposite direction: from Braille to text (Hsu, 2020). The system presented in this paper employs a convolutional neural network model for converting a line of Braille into text; a ratio character segmentation algorithm to enable image segmentation; and optical Braille recognition to convert Braille images into text (Hsu, 2020). AI impact for speech recognition will be in more detail explored in the next subsection.

Non-signers, i.e., people who are not familiar with sign language can communicate with deaf people who speak using the translators of sign language into text or speech (Truong et al., 2016). These translators predominantly use ML algorithms to find the correct sign, like convolutional and recurrent neural networks (Bendarkar et al., 2021) or deep learning (Bantupalli and Xie, 2018). A very promising human-machine interface (HMI) device are communication gloves, which have sensors that interpret the motions of sign languages into natural language combining virtual and augmented reality with AAC (Ozioko and Dahiya, 2022). Ozioko and Dahiya (2022) review many of them, for example, Robotic Alphabet (RALPH), CyberGlove, PneuGlove, 5DT Data Glove and Cyberglove, the last two achieving a recognition accuracy higher than 90%. Apart from purely mechanical interpretation of sign language, several research teams started interpreting facial expressions of people using sign language (Cardoso et al., 2020; Silva et al., 2020). A standard CNN and hybrid CNN+LST were successfully used to translate facial expressions in Brazilian Sign Language Libras (Silva et al., 2020). All these technologies abundantly use almost all the AI algorithms and methods, including NLP essentials, which are their driving force (Cardoso et al., 2020).

Text-to-speech and speech-to-text system preferences and extensions, for example Mercury Reader, Voice Typing and Co-Writer Universal are designed for different operating systems and are compatible with different browsers, including (Dawson et al., 2019). They are frequently used by gifted students who are frustrated due to their dyslexia (Dawson et al., 2019). Mobile applications like ReadandWrite and Speak It! Voice Dream Weaver and libraries, such as Bookshare, Audible are helpful to students with reading and writing disorders like dyslexia (Dawson et al., 2019). They benefit from word prediction too. Word prediction is completely AI powered and it implements various approaches. For example, assistive technology for children with cerebral palsy is based on hidden Markov models (Jordan et al., 2020), a successfully commercialized mobile on-device system that applies deep learning (Yu et al., 2018), whereas context-based word prediction is achieved with naïve Bayes that incorporates latent semantic analyses (Goulart et al., 2018).

Although the effect of AI-based conversational agents on people with disabilities or special needs is rather controversial, they are “widely used to support people services, decision-making and training in various domains” (Federici et al., 2020).

### Voice and speech recognition

As in many other scenarios that involve people with disabilities, AI and various machine learning algorithms show promising results in challenges associated with voice and speech recognition, speech identification, and speech-to-text service applications.

One type of speech disability is the childhood apraxia of speech (CAS), which treatment involves direct therapy sessions with a speech language pathologist. Such sessions must happen during longer periods, which put high demand on time allocation of pathologists. Moreover, many children needing such one-on-one sessions live in rural areas and expenses associated with therapy sessions prevent many children from getting the required support early on Theodoros (2008) and Theodoros and Russell (2008).

Technology in general, and AI and ML in particular, help in enabling children with challenges to receive satisfactory treatment in their home, which makes it a time- and cost-effective solution. One such solution is shown in a study by Parnandi et al. (2013), where a child's progress is assessed through the therapist assigning speech exercises to the child, which then are analyzed using AI algorithms and an assessment is given back to the therapist. In a similar study, further details show how the AI automatically identifies three types of anomalous patterns that are associated with CAS: delays in sound production, incorrect pronunciation of phonemes, and inconsistent lexical stress (Shahin et al., 2015). Especially issues related to measuring the inconsistent lexical stress are addressed using deep neural network-based classification tools (McKechnie et al., 2021). Such a tool is beneficial for both diagnosis and treatment by using the Convolutional Neural Network (CNN) model to identify linguistic units that affect the speech intelligibility (Abderrazek et al., 2020) and voice recognition and production (Lee et al., 2021). The latter study also utilized techniques to acquire bio signals from muscle activity, brain activity, and articulatory activity in order to improve the accuracy.

Deep-learning algorithms are also used with people who stutter, which is a speech disorder that is manifested by an addition of involuntary pauses or repetition of sounds (Sheikh et al., 2021). Using a real-time application, the system records a person's voice, then it identifies and removes stammers by improving speech flow, and then finally produces a speech that is clean from stuttering. The speech flow is improved by implementing an amplitude threshold produced by the neural network model (Mishra et al., 2021).

One type of technology that helps with voice and speech disorders is the STT service. Such services help in maintaining a satisfactory conversation between people living with such disabilities, by capturing their voice and transcribing it into written text that can be read by another person (Seebun and Nagowah, 2020). A form of such disability is also the Functional Speech Disorder (FSD), which is the inability to correctly learn to pronounce specific sounds, such as “s, z, r, l, and th”. Study by Itagi et al. (2019) shows how Random Forest Classifier performs better than other algorithms, such as, Fuzzy Decision Tree and Logistic Regression, when detecting and correcting in real-time FSD cases. These services benefit from the Natural Language Processing (NLP) applied in the STT, which utilizes Google Speech API to convert spoken words into text (Seebun and Nagowah, 2020).

### AI-based assistive technologies and remote education

COVID pandemic was a great challenge for all the students, particularly those who have some communication and learning disabilities. Urgent need to transform traditional in-class education to remote education was an inspiration for many AI researchers to start creating cutting-edge assistive technologies and support massive inclusiveness at all levels of education. AI-based assistive technologies played a significant role in supporting them to learn and study remotely (Zdravkova and Krasniqi, 2021; Zdravkova et al., 2022).

The most widely used operating systems: Windows, MacOS, Android, Linux and Ubuntu provide some or all accessibility features, among which: screen readers, personal assistants, switch controls and voice access and control (Zdravkova et al., 2022).

Learning management systems, such as: Blackboard Ally, Brightspace D2L, Canvas and Moodle have full or partial conformance with WCAG 2.1 (WCAG, 2018). WCAG 2.1, abbreviated from Web Content Accessibility Guidelines version 2.1, is a referenceable ISO technical standard in the form of guidelines and resources that ensures web and mobile accessibility. All LMSs have various embedded screen reader tools, JAWS being common for all, increasing their WCAG 2.1 compliance. Blackboard Ally enables speech recognition via screen reader Read Speaker, while Brightspace D2L uses Dragon Inspection. These two learning management systems provide the opportunity to present learning content with a Braille display (Zdravkova and Krasniqi, 2021).

Video-teleconferencing tools, including the most widely used Zoom, Google Meet, MS Teams, BigBlueButton and Blackboard Collaborate have many features supporting students with motor, vision and hearing impairment. They all incorporate screen reader JAWS, as well as different AI-based plugins (Zdravkova and Krasniqi, 2021).

Massive open online courses (MOOCs), for example Coursera, edX, MIT OpenCourseWare, and OpenLearning offer various accommodations for students with hearing impairments in the form of multilingual subtitles, and transmission of page text toward a Braille display device (Zdravkova and Krasniqi, 2021).

Socially responsible universities in the developed countries have abundantly used most of the assistive features intended for hearing and visually impaired students for decades (Zdravkova and Krasniqi, 2021).

## AI perspective of future assistive technologies

Many children with speech, hearing, and cognitive challenges have limited communication and access to speech-activated gadgets. However, rapidly advancing AI research is opening the way for the creation of new tools to aid in the resolution of these communication issues.

AI has already shown that it has the ability to transform special education and improve results for students with impairments in a variety of ways. Children with ASD who have trouble understanding others' emotions have benefited from AI-driven applications and robots that assist them practice emotion identification and other social skills. Moreover, AI has influenced the creation of algorithms that can aid in the identification of ASD, specific learning difficulties (dyslexia, dysgraphia, and dyscalculia), and attention-deficit/hyperactivity disorder in students (ADHD). For students with disabilities, AI-enhanced therapies have included error analysis to inform instruction and tailored feedback in spelling and maths.

Despite these gains, gaps in AI research for children with impairments, such as AI for students with intellectual and developmental disabilities, remain to be persistent. Because many of these children have numerous disabilities and/or major health concerns, this is an especially vital area of future work. Children with intellectual and developmental disabilities who also have hearing loss or vision impairment, for example, have additional difficulties. Hearing loss and other health difficulties, such as heart issues, are common among Down syndrome students. AI allows for the integration of health data across multiple applications, hence improving the quality of life for these children by promoting independence. This constellation of solutions can aid in the management of student information and the communication of health information among instructors, physicians, and caregivers.

AI algorithms using big data struggle to deal with the individual uniqueness of disabled people (Wald, 2021). There are currently two major issues that prevent the AI use in clinical decision-making in such cases, i.e., a finite amount of labeled data to train the algorithms, and deep neural network models' black-box nature. We believe these issues may be solved in one of two ways. To begin with, self-supervised representation learning has lately been applied to the development of meaningful dense representation from small chunks of data. Furthermore, reinforcement learning paradigms can learn to optimize in any defined environment using an exploration-exploitation paradigm. Second, explainable artificial intelligence methods can be used to enhance the decision-making transparency and trust by creating meta-information that elucidate why and how a decision was reached, while also recommending the factors that influenced the decision the most. This will allow researchers to concentrate on precision in intervention studies and tailored treatment models, while AI algorithms handle the data collection and analysis process.

Moreover, BCI systems for vision impaired people that use steady state visual evoked potentials to stimulate brain electrical activity that enables communication with or without gaze shifting are already a reality. Electronic retinal implants have already restored sight of few patients with degenerative retinal diseases. Cochlear implants successfully provide a sense of sound to hard of hearing and even to deaf people. New brain implants enable people to formulate words and sentences by using their thoughts supporting simple communication. Few years ago, these achievements seemed to be science fiction. With the current pace of technological development, BCI and brain implants that enhance human senses will soon become a reality enabling better inclusivity of people with disabilities.

Nevertheless, every cutting-edge technology is a double-edged sword. To paraphrase Norberg Winner (Bynum, 2017), new technologies, particularly brain implants “may be used for the benefit of humanity”, but they “may also be used to destroy humanity.”

First challenge of cutting-edge communication devices is undoubtedly their rather high price. For example, JAWS and ZoomText and ZoomText Fusion, which are the most widely used accessibility magnifiers and screen readers, have an annual price ranging from 80 to 160 US$ (Zdravkova, 2022). Assistive tools for hearing impaired students AVA and RogerVoice are slightly cheaper, but still not affordable to many (Zdravkova, 2022). On the other hand, the price of DaVinci Pro HD OCR and Logan ProxTalker exceeds 3000 US$, making them available to few highly privileged students (Zdravkova, 2022). If assistive tools are selectively used, they will amplify economic inequality, i.e., the gap between rich and poor. In some wealthier societies, the gender and racial gap might also increase, sacrificing girls and minority groups.

Second challenge is their impact on patients' physical and mental health. Still insufficiently tested communication devices might worsen the state of already feeble hearing or vision sensory organs risking to cause incorrigible deafness or blindness (Shanmugam and Marimuthu, 2021). Such problems might be a result of various reliability problems. This challenge raises the question of liability (Zdravkova, 2019). Although promising, deep brain stimulation reflects the “invasive nature of the intervention” (Cagnan et al., 2019). Another problem related to deep brain stimulation is related to anatomical and pathophysiological differences of people who will undergo the intervention, which can result in inconsistent clinical outcomes (Cagnan et al., 2019).

Next challenge is privacy. Many communication devices are wirelessly connected to medical institutions, either as part of research studies or for health monitoring purposes. The increasing trend of cyber-security threats during COVID pandemic disrupted healthcare institutions worldwide (Muthuppalaniappan and Stevenson, 2021). They affected many hospitals, medical research groups, and healthcare workers, but also the World Health Organization and national authorities of many countries (Muthuppalaniappan and Stevenson, 2021). To avoid data leaks, very strict legal privacy frameworks should be created to significantly increase the level of data protection in public health (WHO, 2021).

Final challenge is related to the social acceptability of new technologies (Koelle et al., 2018). According to this research novel technologies and applications “might create new threats, raise new concerns and increase social tension between users and non-users” (Koelle et al., 2018). Many societies are technology skeptical and their first reaction to cutting-edge technologies is full resistance. If officially approved, there will be many disabled people who will be concerned with their impact making a vicious circle, which might worsen the situation instead of improving it.

In order to avoid all the challenges mentioned above, innovation in research should be very responsible. All potential ethical and legal challenges should be anticipated on time, and their remedies should be carefully included into new cutting-edge assistive technologies by design.

## Author contributions

KZ, VK, FD, and MF: conceptualization, formal analysis, investigation, and writing–original draft preparation. KZ and FD: methodology, writing–review and editing, and project administration. KZ: supervision. All authors have read and agreed to the published version of the manuscript.

## Conflict of interest

The authors declare that the research was conducted in the absence of any commercial or financial relationships that could be construed as a potential conflict of interest.

## Publisher's note

All claims expressed in this article are solely those of the authors and do not necessarily represent those of their affiliated organizations, or those of the publisher, the editors and the reviewers. Any product that may be evaluated in this article, or claim that may be made by its manufacturer, is not guaranteed or endorsed by the publisher.
